# Improving the Quality of Life of Family Caregivers of People with Alzheimer's Disease through Virtual Communities of Practice: A Quasiexperimental Study

**DOI:** 10.1155/2021/8817491

**Published:** 2021-04-05

**Authors:** Montse Romero-Mas, Anna Ramon-Aribau, Dyego Leandro Bezerra de Souza, Andrew M. Cox, Beni Gómez-Zúñiga

**Affiliations:** ^1^Universitat de Vic-Universitat Central de Catalunya (UVic-UCC), Spain; ^2^Federal University of Rio Grande do Norte, Brazil; ^3^University of Sheffield, UK; ^4^Universitat Oberta de Catalunya, Spain

## Abstract

Caring for a person with dementia burdens family caregivers, and there is a close negative relationship between this burden and their quality of life (QoL). Research suggests that caregivers' main needs are information and training about the disease and support from others experiencing the same situation, and Internet interventions hold considerable promise for meeting these needs. Virtual communities of practice (VCoPs) are Internet frameworks to share knowledge where members collaborate and achieve a sense of trust in the community. This paper seeks to evaluate the impact of participating in a VCoP (developed through an App) on the QoL of caregivers to people with Alzheimer's. Results show QoL before and after the intervention changed significantly. The impact of VCoP on caregivers' overall QoL is moderated by age and relation with the person with Alzheimer's, specifically those over 65, and spouses. VCoPs allow interaction and knowledge sharing among caregivers which provide them mainly with information and support from peers helping them to meet their needs. Furthermore, caregivers' QoL did not decrease when their relative deteriorated functionally, which could be due to the participation in VCoP. Although we found significant pre- and post differences in caregivers' health literacy, we must report the ambiguous result that this variable only impacts on QoL's physical domain. Participants also reported that they had a positive experience because the App was perceived to be a useful tool, because they could manage their own participation and they met peers and felt less lonely. Results suggest that participation in a VCoP impacts positively on caregivers' QoL.

## 1. Introduction

Because of the global rise in life expectancy, the impact of chronic conditions associated with age is growing. One of the most challenging issues is dementia, and 50% of dementia cases are a result of Alzheimer's disease [1]. Today, dementia is a major public health problem. 47 million people worldwide live with dementia, and this figure is expected to increase to 75 million by 2030 and 132 million by 2050 [2]. Within this context, the role of family caregivers is of increased importance (hereafter, “caregivers”) [3].

It is well established that caregivers suffer from physical strain, increased sense of burden, psychological stress, depression, and anxiety [4]. The literature also suggests that there is a close negative relationship between these effects and quality of life (QoL). It is a fact that caregivers' QoL is related to that of the care recipient's [5]. Although the ability to improve the general QoL of life of caregivers appears to be limited, there is evidence that specific components of QoL, such as burden, mood, and perceived stress, are responsive to interventions [6]. Research suggests that caregivers' main needs are information and training about the disease and support from others experiencing the same situation [7, 8]. Social support interventions provide a venue for caregivers to share their caregiving experience, to build social relationships and to receive emotional comfort and informational material [9]. Finally, some researchers suggest that when studying family caregivers, demographic factors and caring characteristics should be considered: age, gender, marital status, education, time spent in caring, or the physical intensity of affection of people with Alzheimer's disease [9, 10].

To deliver appropriate support to caregivers, communities of practice (CoPs) could be an option. The literature offers a rich field of evidence of how learning and information sharing can happen in a community context. A CoP is “a group of people who may not normally work together but who are acting and learning together in order to achieve a common task whilst acquiring and negotiating appropriate knowledge” [11]. Considering this definition, the CoP model is one way in which a legitimate form of group working may be used to explore health and social care issues and develop practice [11]. In line with this suggestion, virtual communities of practice (VCoPs) could be a potential Internet-based intervention. VCoPs are virtual communities rooted in community of practice theory [12]. VCoPs are frameworks for a form of social group who share knowledge where members establish a culture of collaboration, and they ultimately come to have a sense of trust in the community [13]. Greater levels of participation in them help to share knowledge, disseminate ideas quickly, and provide emotional support among members [14]. Furthermore, a virtual community is dynamic and person-centred and so could help to improve healthcare outcomes for people with chronic disease [15]. The authors of this paper intend to seek to gather evidence that this concept could be beneficial for caregivers. Therefore, it has to be considered that benefits of the health VCoP could be to increase interaction among the members; knowledge creation and information sharing; peer, social, and emotional support, monitor public health, and ultimately to influence health policy [16].

We cannot forget that VCoPs are virtual environments, and that is why we must also consider the effect of the medium. Recent research has found an association between eHealth literacy and health literacy level with carers' perceptions about their caregiving role, self-efficacy, and coping strategies [17]. eHealth literacy has been defined as “the ability to seek, find, understand, and appraise health information from electronic sources and the extent to which individuals have context-specific and analytical skills needed to successfully navigate online health information” [18]. The benefits of eHealth use, defined as the usage of health services and information disseminated through the Internet and related technologies [19], are potentially very important among caregiver populations [20]. Thus, Internet interventions hold considerable promise for meeting the educational and support needs of dementia caregivers [21].

To summarize, (i) caregivers have their QoL diminished by their situation, (ii) their unmet needs have to do with information and support, and (iii) virtual environments seem to be a means to the fulfilment of these needs. Thus, the purpose of this study is to describe the relation between the QoL of the family caregivers of a person with Alzheimer's disease and their participation in a VCoP. Our main hypothesis is that VCoPs may help caregivers to reach their unmet needs and, consequently, improve their QoL. The literature suggest that we must consider how demographic factors (such as age, gender, number of offspring, and level of education and marital status) together with the caring characteristics may influence caregivers' QoL. We presume VCoPs could neutralise some of the negative effects for caregivers' QoL related to demographic and caring characteristics. We should also consider that diseases evolve, so we seek also to investigate the effect of the functional deterioration of the person with Alzheimer's on caregivers' QoL while participating in a VCoP. As we have said, we opted for a virtual environment for this study so we need also to see the impact of caregivers' eHealth literacy on the effects on QoL of their involvement in a VCoP. We hypothesise that caregivers with greater eHealth literacy would improve their QoL more while participating in a VCoP than those with a lower level of literacy.

## 2. Materials and Methods

### 2.1. Participants and Procedure

Study participants were family caregivers of people having Alzheimer's disease. All participants were recruited between July 2017 and April 2018 in the Osona region of Catalonia (Spain). In order to recruit the caregivers for the study, researchers first shared the project idea with Osona's Association of Alzheimer's Family Caregivers (AFMADO). Then, it was disseminated to the hospital healthcare system and community health and social fields of the whole Osona region. In total, five explanatory sessions with caregivers (individual and group) and twelve sessions with health professionals (individual and group) were held. From these sessions, the researchers were able to recruit 38 caregivers. The inclusion criteria were that participants should be a family caregiver of a person with Alzheimer's; to have Internet access; to be able to use a smartphone, tablet, or computer to download the App; and to have their relative living in Osona. The exclusion criteria were caregivers who did not want to participate in the investigation and caregivers who did not own an email address (at it was required to get the App installed).

A pretest-posttest quasiexperimental design was adopted in this study, in which a total of 38 participated in the pretest and 37 participated in the posttest. The participants were randomly divided into two groups: the first group consisted of 19 caregivers plus an expert caregiver and the second group consisted of 19 plus 3 health professionals. The communities were built using an App designed mainly for this project. Access to the App was exclusively for participants. The communities were active from the 24^th^ April 2018 to the 20^th^ February 2019.

### 2.2. Intervention

**Figure 1:**
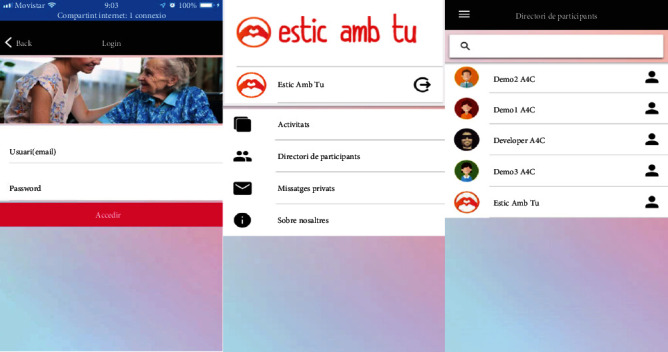
Samples of the App “Estic amb tu—I'm With You” screenshots.

The intervention was an Internet-based tool with a design based on CoP theory. The technology chosen to establish the VCoP was a mobile application (App). First, features of an ideal online social support network were derived from the literature: to have a secure synchronous communication option, intuitive user interface, trusted or moderated content, mobile access, and data sharing [22]. Then, the App “Estic amb tu - I'm With You” was designed to offer such features, including within its functions a conversation space, a directory of members with information about each of them, community management tools, and so forth [12] (see Figure 1).

Specifically, we followed the VCoP model developed for a study of “Wikipedia,” paying attention to the six elements they considered: individuals, practice, content, interactions, community, and technology [23]. With the aim of helping the groups to construct knowledge through shared learning, moderators were introduced to the VCoP from the beginning [24]. One of the two communities was moderated by an expert caregiver whereas the other by three health professionals (a nurse, a geriatric physician, and a psychologist). Administrative and technology aspects were handled by the researchers. Three face-to-face sessions were facilitated for the members. Access to the App and to these sessions was restricted to study participants. The first support session was conducted once the App was available to download. It mainly dealt with the download and use of the App, together with the introduction of participants. The goals of the second meeting were socializing, provoking debates that would follow later in the virtual forum, and discussing difficulties that might hinder the work of the community. The last face-to-face meeting was for evaluation and closing the App. All the face-to-face sessions were held at the University of Vic-Central University of Catalonia (UVic-UCC) led by a researcher of the project. If any participant could not attend the meeting, researchers provided individual feedback to them.

The two communities were developed independently through the App within their own practice (the specific knowledge the community shared, developed, and maintained). Resource limitations meant that the community could not be continued after a period of 10 months. On joining, participants were advised that there was a time limit to the trial, though the precise date for this was not determined until later in the project. The same day of closing the App, researchers sought feedback from participants in order to having more data for the intervention evaluation.

### 2.3. Data Handling

Caregivers' QoL shortly before starting the VCoP intervention and shortly after was measured for each participant. Living together with a person with Alzheimer's may not necessarily be associated with a lower QoL in all areas of life. QoL was considered as a multidimensional concept [25]. We used the Spanish version of WHOQOL-BREF [26], a self-administered questionnaire to assess QoL which subdivides QoL into four subdomains (physical health, psychological health, social relationships, and environment). All the other parameters studied in our research were moderator variables. Demographic and caring variables were collected about the participants' preintervention. In addition, we measured the functional deterioration of the person with dementia with the Spanish version of the Barthel index [27]. This test was answered by the caregiver participants regarding their care recipient pre- and post intervention. In addition, in order to measure the eHealth literacy of the participants, we used the Health Literacy Scale (eHEALS), again pre- and post intervention [18].

Finally, during the last face-to-face session, feedback about the intervention was requested from the caregivers through a survey. This survey had two open-ended questions to evaluate the intervention: “How do you assess the experience of participating in the VCoP?” and “Would you like to be part of another VCoP if we consider activating a second version?”

### 2.4. Data Analysis

We used SPSS 23.0 for all the quantitative data analyses. All the analyses are bivariate as we contrasted two variables during the analyses. The confidence level was established at 95%. First, we compared the demographic factors and caring characteristics of the caregivers to investigate their influence in their QoL. Then, we compared the pre- and post values to draw conclusions about the impact on QoL of caregivers to people with Alzheimer's while participating in a VCoP, the functional deterioration of care recipients, and caregivers' change in eHealth literacy. Finally, we determined the empirical relationship between all the variables with QoL to evidence predictor and moderator variables.

Data from the survey was analysed through both thematic analysis and descriptive analysis. Thematic analysis is best suited to elucidating the conceptualizations that a given group holds on a topic and also fits the research questions focused on exploring the caregiver's experiences [28]. As the first question was open ended, thematic analysis was used to identify themes. Then, descriptive analysis for the second one was carried out, as it was a binary variable (yes or no).

### 2.5. Ethics

All participants included in the study met the inclusion criteria. Participants signed an informed consent form. Ethics approval was obtained from the University of Vic-Central University of Catalonia Ethics Committee.

## 3. Results

Given that we had the participants distributed into two VCoPs, one without (VCoP1) and one with health care practitioners included (VCoP2), the first step was to test whether this variable had any impact on outcomes. We identified no significant statistical differences between them considering their QoL, demographic variables, caring characteristics, functional deterioration of the person with Alzheimer's, and eHealth literacy (see Tables 1 and 2).

Consequently, the two groups can be considered statistically comparable, and results of this project set out below are able to be presented about all participants as a whole.

### 3.1. Descriptive Statistics

We included 38 caregivers in the VCoPs. The youngest participant was 28 years old, while the oldest was 81, and they had an average age of 56 years. 29 (79%) were female and 8 (21%) male. 27 (73.7%) were married, 6 (15.8%) divorced, and 4 (10.5%) single. 29 (78.9%) were offspring of the recipient of care, 5 (13.2%) spouses, and others 3 (7.9%). These characteristics are consistent with results from other national studies involving caregivers for elderly people [29]. They had a mean of 1.5 offspring. In terms of educational attainment, 7 (18.4%) had reached primary studies, 17 (47.4%) secondary, and 13 (34.2%) university studies. Whereas the mean of length of caregiving was 4 years, the length of time varied from 2 to 8 years.

### 3.2. Analysis

With the aim of evaluating the impact on the QoL of caregivers of people with Alzheimer's disease while participating in a VCoP, we compared the mean of WHOQOL-BREF questionnaires pre- and post intervention. The initial mean of QoL was 66.65 (out of 100), while after caregivers participated in the VCoP, this rate increased to 69.50. (we had one individual drop out). Paired sample *t* tests suggests that caregivers' QoL before and after the intervention had changed significantly (see Table 3).

This means that caregivers increased their overall QoL while participating in the VCoPs, albeit there were no differences when we focused on physical, psychological, social, or environment domains individually. After this, we examined the care recipient's functional deterioration pre- and post intervention using the Barthel index. The original Barthel index mean was 66.84 whereas the final mean was 59.86. The Wilcoxon test indicates that this change is statistically significant (see Table 3). Finally, we contrasted caregivers' eHealth literacy before and after participating in the VCoP through eHEALS. The starting eHEALS mean rate was 26.10 (out of 40), whereas the rate was 30.68 at the end of the study. The Wilcoxon test again shows that this difference is statistically significant (see Table 3).

At this point, we introduced the variable “QoL_change” for all the participants. In fact, there were five variables as we studied the change of overall QoL together with the change of its four domains for each participant. These variables represented the change between pre- and post intervention. Hereafter, when mentioning QoL, we will refer to it as “QoL change.”

Then, we explored the influence of caregivers' demographic variables on their QoL: age, gender, level of education, and marital status and number of offspring. The age parameter was turned to a qualitative variable having a value of 0 to 65 years or more than 65. From these five qualitative demographic variables, a significant difference in age could be found with caregivers' QoL. A Mann-Whitney *U* test shows there was a significant difference between age groups in terms of their overall rate of QoL. The participants who improved their QoL more were the ones over 65 as their overall QoL mean increased from 66.3 to 74.64, whereas the participants up to 65 years old increased their QoL from 66.70 to 67.85. Specially, there was found to be an association with the psychological domain of QoL (see Table 4). None of the other demographic variables saw statistically significant differences (see Table 4).

Regarding caring characteristics, the variables we studied were the familial relation with the person with Alzheimer's, length of caring, and functional deterioration. With respect to the relation with the person with Alzheimer's (spouse, offspring, and others), the Kruskal-Wallis test showed that there was a statistically significant difference with the overall QoL rate (see Table 4). Specifically, if the carer was a spouse, their QoL improved more as the mean went from 69.75 to 75.68. However, offspring increased their overall QoL less, and “other” relatives decreased their overall QoL from 90.75 to 87.50. A relation with the psychological domain was found with again more influence on caregivers who were spouses of the care recipient (see Table 4). There was a significant negative correlation between length of caring and caregivers' QoL regarding the psychological and social domains (see Tables 4 and 5). Nevertheless, the Spearman correlation test found that there was no correlation between care recipients' functional deterioration and caregivers' QoL (see Tables 4 and 5). Even though the Barthel index decreased, caregivers' QoL did not decrease. Finally, the Spearman correlation demonstrated a positive correlation between eHEALS with the physical domain of QoL (see Tables 4 and 5).

**Figure 2:**
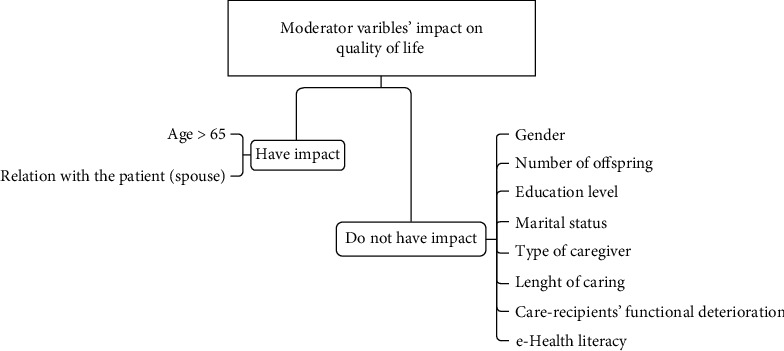
Impact of VCoP on caregivers' overall quality of life: moderator variables.

For the purpose of having visual perspective of the impact of VCoP on caregivers' overall QoL, the researchers provide the following Figure 2.

Finally, 19 participants gave feedback on the intervention through the 2-question survey. Participants reported that they had a positive experience because the App was perceived to be a useful tool, because they could manage their own participation and they met peers and felt less lonely. They also asked about continued use of the App. They reflected that because they were in different phases of care, this sometimes reduced their ability to share information (see Tables 5 and 6).

Then, when we asked the participants if they would be interested in participating in another VCoP, 69.5% of participants answered positively, whereas 30.5% of participants gave a negative answer.

## 4. Discussion

The literature indicates that Internet interventions hold considerable promise for meeting the educational and support needs of family dementia caregivers at reduced cost [21]. Confirming this, the results of our research showed that caregivers improve their QoL while participating in a VCoP. Our study has investigated both the impact of VCoP on global QoL scores and on its domains. Caregivers increased their QoL while participating in a VCoP further suggesting that Internet interventions may help caregivers reach their unmet needs.

In prior research on caregivers to those with chronic disease, demographic parameters were associated with caregivers' QoL. Specifically, the most common related factors were gender, age, and level of education [30, 31]. Female caregivers, typically the majority of caregivers, appear to face a greater adverse impact on QoL [32]. In our study, probably due to the lack of variability as we had 79% of female caregivers, no significant difference was found between males and females in their QoL. In our research, age was the only demographic parameter which impacted caregivers' QoL. Interestingly, even though the literature states older people may be more vulnerable to deterioration in their QoL while caring [33], we found that the oldest caregivers were the ones improving their QoL the most, specifically their psychological QoL. With the other demographic factors studied, no significant differences could be found.

With reference to the characteristics of caring, previous literature indicates that the longer a caregiver remains in his or her role, the more likely negative outcomes are to occur [33]. In addition, literature suggests that hours spent offering care have a significant relationship with the QoL of chronic illness caregivers [34]. Aligned with the literature, within our study, we noted a negative correlation between “length of caring” and psychological and social domain of QoL.

Moreover, previous literature found a significant association between the “relation with the person with Alzheimer's” variable and caregivers' QoL [30]. Our research agrees with the results found in prior studies as there was such a difference between the relation with the person with Alzheimer's (spouse, offspring, and others) and overall QoL. However, our data indicates that spouse was the category that improved their QoL most and suggests that the VCoP was helpful for them. In addition, as in previous research [30], there was an association between relation with the person with Alzheimer's and the psychological domain of QoL, again with more influence in spouses. Many family members find meaning in providing care to a loved one, feel more useful, gain new skills, and experience other benefits from giving back to those who have helped them in the past [35]. Literature states that those who live with a care recipient tend to be a spouse or a family member, provide more hours of caregiving, feel more responsible for caregiving tasks as part of their familial duties, and experience the greater physical and emotional closeness of the care recipients [36, 37]. However, the positive impact of VCoP was shown as spouses increased their QoL the most.

There is evidence in existing research that the caregivers' QoL gets worse when the functional capacity of the elderly person with Alzheimer's disease declines [38]. Additionally, when care recipients have moderate/severe dementia symptoms such as frequent distressed behaviours, there seems to be more potential opportunities to improve the caregiver QoL [39]. In the current study, no correlation between the functional deterioration of the person with Alzheimer and caregivers' QoL was found. Nonetheless, caregivers' QoL did not decrease when their relative had deteriorated functionally. Hence, the relation between the functional deterioration of the person with Alzheimer's and caregiver's QoL could be altered for many reasons, among them the participation in VCoP.

Prior studies suggest that the level and the role of health literacy among carers of people with dementia are very limited. In our study, the rates of initial eHealth literacy were already high before the intervention (26.34 out of 40). Previous investigations have pointed to an association between eHealth use with lower levels of social functioning, communication, worry, and family relationship. In this study, eHealth literacy impacted positively on the physical domain of the caregivers' QoL, in a way contrasting with findings from the existing literature. Still, it has to be taken into consideration that participants' mean age was over 55 years, and among this population, eHealth literacy is a rather underresearched concept [20].

The encouraging feedback from some participants together with their interest in repeating the experience is consistent with the main findings of this study offering qualitative support to the value of a VCoP. In summary, it could be recommended to consider VCoP to enhance family caregiver QoL. Improved caregivers' QoL can raise well-being for caregivers and may, in turn, raise the quality of people with Alzheimer's disease care. Results confirmed that participation in a VCoP impacts positively on caregivers' QoL. Earlier research already suggested greater levels of participation in virtual communities can help to share knowledge, disseminate ideas quickly, and provide emotional support among members [14]. Finally, in previous literature, low caregiver health literacy was associated with increased caregiver burden and increased health service use [40].

Nevertheless, our study presents limitations. Despite using a range of strategies, the recruitment for this study was difficult. These difficulties often occur in Internet-based intervention studies [41], suggesting it may be due to caregivers' attitudes toward these programs [42]. However, literature describes caregivers' reluctance to participate in face-to-face services too [43]. The small sample, together with the caregivers' characteristics, where most of caregivers are women, spouses, or offspring of the dementia sufferer and married [29], could be a barrier when seeking for associations between moderator variables and QoL. In addition, this study produced evidence that older caregivers experienced a positive impact while younger caregivers were more negatively influenced. Clearly, the age variable should be further explored as additional family burden could have influenced this effect. As well, in our research, there were several moderating factors from both caregivers and caring which the study did not consider. Caregivers' physical condition or financial caregivers' situation are aspects which literature points to having a potential influence on caregivers' QoL [44]. A future line of research would be to explore the levels of participation and the content of the interaction performed in the VCoP, specifically in relation with caregivers' QoL. Moreover, there is still little research on the health literacy of carers. Research in this area would be timely.

## 5. Conclusions

As we have seen in previous sections, our main hypothesis has been confirmed: caregivers can benefit from a VCoP. VCoPs enable interaction and knowledge sharing among caregivers which provide them mainly with information and support from peers helping them to reach their needs. VCoP can neutralise some of the negative effects for caregivers' QoL related to demographic and caring characteristics. The impact of VCoP on caregivers' overall QoL is moderated by age and relation with the person with Alzheimer's. Specifically, those over 65 were found to benefit more which is in contrast with existing literature, and spouses also benefit, which is in line with the literature. VCoPs allow interaction and knowledge sharing among caregivers which provide them mainly with information and support from peers helping them to reach their needs. Furthermore, caregivers' QoL did not decrease when their relative deteriorated functionally which could be due to many reasons, among them the participation in VCoP. Although we found significant pre- and post differences in caregivers' health literacy, we must report the ambiguous result that this variable only impacts on QoL's physical domain. Participants also reported that they had a positive experience because the App was perceived to be a useful tool, because they could manage their own participation and they met peers and felt less lonely.

## Figures and Tables

**Table 1:** Statistical differences between demographic and caring values of the two groups.

	VCoP1	VCoP2	*p* value
Gender^a^, *n* (%)			0.693
Male	3 (15.78)	5 (26.32)	
Female	16 (84.22)	14 (73.68)	

Marital status^b^, *n* (%)			0.667
Married	15 (78.94)	13 (68.42)	
Single	2 (10.55)	2 (10.53)	
Divorced	2 (10.55)	4 (21.05)	

Level of studies^c^, *n* (%)			0.366
Primary	4 (21.05)	3 (15.78)	
Secondary	10 (52.63)	8 (42.11)	
University	5 (26.32)	8 (42.11)	

Relation/person with Alzheimer's^b^, *n* (%)			0.050
Offspring	12 (63.17)	18 (94.74)	
Spouse	4 (21.05)	1 (5.26)	
Other	3 (15.78)	0 (0)	

Age^d^, *M* (SD)	56.31 (12.06)	55.15 (9.22)	0.492
Offspring number^d^, *M* (SD)	1.71 (0.99)	1.36 (1.01)	0.253
Length of caring^d^, *M* (SD)	3.26 (2.21)	4.78 (2.63)	0.056

^a^Exact-test de Fisher; ^b^Pearson's chi-square; ^c^chi-square linear tendency; ^d^*U* de Mann-Whitney.

**Table 2:** Statistical differences between caregivers' WHOQOL-BREF, Barthel, and eHEALS preintervention of the two groups.

Variable	VCoP1	VCoP2	*p* value
WHOQOL_BREF pre^a^, *n* (*R*)	19 (21.32)	19 (17.68)	0.313
Barthel pre^a^, *n* (*R*)	19 (22.26)	19 (16.74)	0.122
eHEALS pre^a^, *n* (*R*)	19 (19.24)	19 (19.76)	0.884

^a^Mann-Whitney *U.*

**Table 3:** Statistical differences between caregivers' WHOQOL-Barthel-eHEALS pre- and postintervention.

Variable	*n*	Mean	SD	Median	*p* value
WHOQOL-BREF					
Overall-Pre	38	66.60	14.60	65.75	0.002^∗^
Overall-Post	37	69.50	13.90	72.00	
Physical-Pre	38	69.78	17.37	69.00	0.307
Physical-Post	37	70.67	18.48	75.00	
Psychological-Pre	38	63.86	18.84	63.00	0.426
Psychological-Post	37	64.83	21.38	69.00	
Social-Pre	38	68.05	17.41	69.00	0.364
Social-Post	37	68.48	20.64	75.00	
Environmental-Pre	38	65.64	15.32	69.00	0.615
Environmental-Post	37	64.86	17.49	63.00	

Barthel					
Pre	38	66.84	32.74	75.00	<0.001^∗∗^
Post	35	59.85	32.95	65.00	
eHEALS					
Pre	38	26.10	8.22	26.00	<0.001^∗∗^
Post	35	30.68	7.56	30.00	

**Table 4:** Statistical differences between qualitative caregivers' variables and QoL.

Variable	*N*	Mean diff. overall QoL_change	SD overall QoL_change	Overall QoL *p* value	Physical QoL *p* value	Psycho. QoL *p* value	Social QoL *p* value	Environ. QoL *p* value
Age	37			0.025^∗^	0.566	0.008^∗^	0.270	0270
≤64	28	1.08	15.84					
>65	9	8.33	6.48					
Gender	37			0.479	0.148	0.094	0.0957	0.871
Male	8	7.53	13.60					
Female	29	1.54	14.57					

Level education	37			0.760	0.119	0.153	0.907	0.999
Primary	7	6.14	8.83					
Secondary	17	4.39	14.54					
University	13	-0.94	16.62					

Marital status	37			0.092	0.440	0.633	0.285	0.225
Married	27	4.06	11.63					
Single	4	-14.02	26.11					
Divorced	6	8.70	10.08					

Offspring number	37			0.415	0.358	0.255	0.684	0.832
None	8	-4.18	21.79					
One	7	8.29	13.50					
Two	16	4.66	11.57					
Three	6	0.29	7.67					

Relation/person with Alzheimer's	37			0.045^∗^	0.132	0.042^∗^	0.918	0.292
Offspring	29	2.98	15.69					
Spouse	5	7.80	4.78					
Other	3	-6.75	6.24					

**Table 5:** Correlations between quantitative caregivers' variables and QoL.

Variable	*N*	Mean diff. overall QoL change	SD overall QoL_change	Overall correl. coeff. QoL	Physical correl. coeff. QoL	Psycho. correl. coeff. QoL	Social correl. coeff. QoL	Environ. correl. coeff. QoL
Length of caring (years)	37	4.19	2.60	-0.237	-0.176	-0.372^∗^	-0.446^∗^	-0.171
Change_Barthel	35	-9.67	13.22	-0.750	-0.323	-0.069	-0.282	0.123
Change_eHEALS	35	4.45	7.58	0.256	0.431^∗^	0.148	0.134	0.082

**Table 6:** Themes and quotations.

Theme	Quotation
Positive experience	“The group is positive, for me it has been very good” (participant1) “I really enjoyed the App” (participant2)
Another tool	“This App adds to the range of options available” (participant3) “One more tool to look for support apart from professional care which is very good” (participant4)
Managing own participation	“I am not active. When there were no messages, I missed it” (participant5) “The key is that if you feel like speaking, you speak and if you don't, you don't speak” (participant 6)
Peer-people	“Being able to talk to people who have the same thing as you, the same problem as you and you see that we can't do anything about it because the disease advances” (participant7) “Because of all I read I realise what's to come, this has accelerated my learning and told me to take advantage, take advantage now that you can do all these things” (participant8)
App continuity	“I wish the App could continue; it shouldn't be closing down” (participant7) “I'd like to know if there was a chance that keep the App alive and that people could be added” (participant1)
Availability	“You can enter whenever you want” (participant9) “Advantage of doing it when you want” (participant10)
Different phases	“Having caregivers going through different emotional and disease points is an advantage and a disadvantage” (participant6) “We are all at different phases, emotionally and regarding to the disease” (participant11)
No loneliness	“With this App you have people around you who listen to you and love you” (participant11) “You don't feel alone anymore” (participant13)

## Data Availability

The data used to support the findings of this study have not been made available due to privacy/ethical restrictions.
